# Expanding the repertoire of Antibody Drug Conjugate (ADC) targets with improved tumor selectivity and range of potent payloads through in-silico analysis

**DOI:** 10.1371/journal.pone.0308604

**Published:** 2024-08-26

**Authors:** Umesh Kathad, Neha Biyani, Raniero L. Peru y Colón De Portugal, Jianli Zhou, Harry Kochat, Kishor Bhatia

**Affiliations:** 1 Lantern Pharma Inc., Dallas, TX, United States of America; 2 The University of Tennessee Health Science Center, Memphis, TN, United States of America; Icahn School of Medicine at Mount Sinai Department of Pharmacological Sciences, UNITED STATES OF AMERICA

## Abstract

Antibody-Drug Conjugates (ADCs) have emerged as a promising class of targeted cancer therapeutics. Further refinements are essential to unlock their full potential, which is currently limited by a lack of validated targets and payloads. Essential aspects of developing effective ADCs involve the identification of surface antigens, ideally distinguishing target tumor cells from healthy types, uniformly expressed, accompanied by a high potency payload capable of selective targeting. In this study, we integrated transcriptomics, proteomics, immunohistochemistry and cell surface membrane datasets from Human Protein Atlas, Xenabrowser and Gene Expression Omnibus utilizing Lantern Pharma’s proprietary AI platform Response Algorithm for Drug positioning and Rescue (RADR^®^). We used this in combination with evidence based filtering to identify ADC targets with improved tumor selectivity. Our analysis identified a set of 82 targets and a total of 290 target indication combinations for effective tumor targeting. We evaluated the impact of tumor mutations on target expression levels by querying 416 genes in the TCGA mutation database against 22 tumor subtypes. Additionally, we assembled a catalog of compounds to identify potential payloads using the NCI-Developmental Therapeutics Program. Our payload mining strategy classified 729 compounds into three subclasses based on GI_50_ values spanning from pM to 10 nM range, in combination with sensitivity patterns across 9 different cancer indications. Our results identified a diverse range of both targets and payloads, that can serve to facilitate multiple choices for precise ADC targeting. We propose an initial approach to identify suitable target-indication-payload combinations, serving as a valuable starting point for development of future ADC candidates.

## Introduction

Antibody-drug conjugates (ADCs) offer a promising approach towards targeted cancer treatments. The approval of 12 ADCs for treatment of hematological and solid tumors, along with more than 170 novel ADCs in clinical development, serves as compelling evidence of the growing acceptance of this therapeutic approach in treating cancers [1].

ADCs leverage the specificity of antibodies and increasingly innovative linker-payload technologies to deliver potent cytotoxic agents selectively to tumor cells, while minimizing the adverse effects to healthy cells. The efficacy and safety of ADCs is determined by interplay of each of its three essential components: an antibody, a cytotoxic payload, and a chemical linker [2].

While ADCs have demonstrated remarkable success as targeted therapeutics, there are still challenges to be addressed. For selective targeting and improved efficacy of ADCs it is highly desired to: 1) Optimize target selection which plays a pivotal role in the establishment of a therapeutic window, 2) identification of highly potent payloads with diverse mechanisms of action capable of selective targeting [3] and a 3) linker to effectively transport the payload, either by releasing or retaining it [4].

The range of targets currently undergoing clinical investigation is narrow with, notable focus on a few antigens such as HER2, Trop-2, CLDN18.2 and EGFR [5], frequently leading to clinical benefit for a limited set of cancers. The optimal target for ADC development should exhibit both high and uniform expression in tumor cells, while excluding expression in normal cells [6]. ADC targets currently under development represent a wide-ranging expression profile in both tumor and normal cells. In addition, expression of the target antigens is often modulated in accordance with the mutation profile of tumor cells [7, 8]. Therefore, in the pursuit of next-generation ADCs, it is crucial to take into account the uniformity of target expression among patients who are positive for the target, along with the exploration of novel targets [9].

The payload is another key component of ADC, which is frequently composed of highly potent cytotoxic agents with IC_50_ values ranging from picomolar to low nanomolar ranges [10]. Microtubule targeting agents and DNA damaging agents are among the most commonly used payloads representing 57% and 17% of clinically tested ADCs, emphasizing the scarcity of diversity in terms of mechanism of action [11]. Furthermore, these payloads frequently encounter issues related to toxic side effects, emergence of drug resistance, and efficacy against a limited range of tumor targets [10,12]. There is a need for the proficient alignment of the payload’s mechanism of action with the biological characteristics of the target tumor biology [13]. Identification of payloads with high potency, selective targeting, and diverse mechanisms of action capable of evading drug resistance is highly desired for enhancing ADC’s effectiveness [10].

In our present study, we aimed to uncover ADC targets and payloads with improved tumor selectivity. To select target candidates for ADCs, we implemented the initial steps outlined in the approach presented by Razzaghdoust A et al. [14] for ADC target identification. Our present work distinguishes in the subsequent research methodology and steps by including a comparative analysis of expression levels using datasets from IHC staining, RNAseq followed by GEO study (GSE42519) [15] and mutational profiles. We utilized Lantern Pharma`s proprietary AI platform RADR^®^ (Response Algorithm for Drug positioning and Rescue) and the Human Protein Atlas (HPA) database version 22.0 (https://v22.proteinatlas.org/) to integrate transcriptomics, proteomics, immunohistochemistry (IHC) from 20 tumor types and 44 normal tissues, as well as cell surface membrane based datasets [16]. Elevated levels of the target antigen on blood cell types can impede the accumulation of ADCs at the tumor site [8]. Therefore, in the subsequent stage, we utilized the data from the GEO study (GSE42519) [15] to eliminate the targets that display high expression across various blood cell types, such as hematopoietic stem cells (HSCs) and multipotent progenitor cells (MMPs). Furthermore, we employed the TCGA mutation database to explore the impact of altered genes in several tumor types on the expression levels of targets, aiming to improve precision targeting of ADCs for specific patient populations.

To identify potential payloads with selective tumor targeting, we employed the NCI-DTP data, which has screened over 50,000 molecules utilizing a 60 tumor cell line screening platform over the span of 20 years [17]. We primarily focused on the compounds exhibiting activity at picomolar (< = 1nM) and low nanomolar (>1nM - 10nM) range in 9 cancer indications covered by NCI60 cell lines. In the current study, we report a strategy to compile a list of compounds that demonstrate specific or heightened sensitivity towards the desired cancer type. This approach can potentially aid in identification of novel payloads, as well as the possibility of the repurposing of existing cytotoxic agents in a tumor selective manner.

Notably, a recent article published by bosi et al., 2023 [6] made valuable contributions by investigating ADC targets and potential predictors of treatment response across multiple cancer types. In considering the comparison, it becomes evident that while their work focused on clinically developed targets and payloads, our research contributes towards identification of novel targets and unexplored potential payloads as well.

We examined an initial approach to explore target, indication and payload combination. This may serve as a good starting point for further investigations and refinements in the complex process of ADC design.

## Results

### Identification of potential ADC target candidates

**Fig 1:**
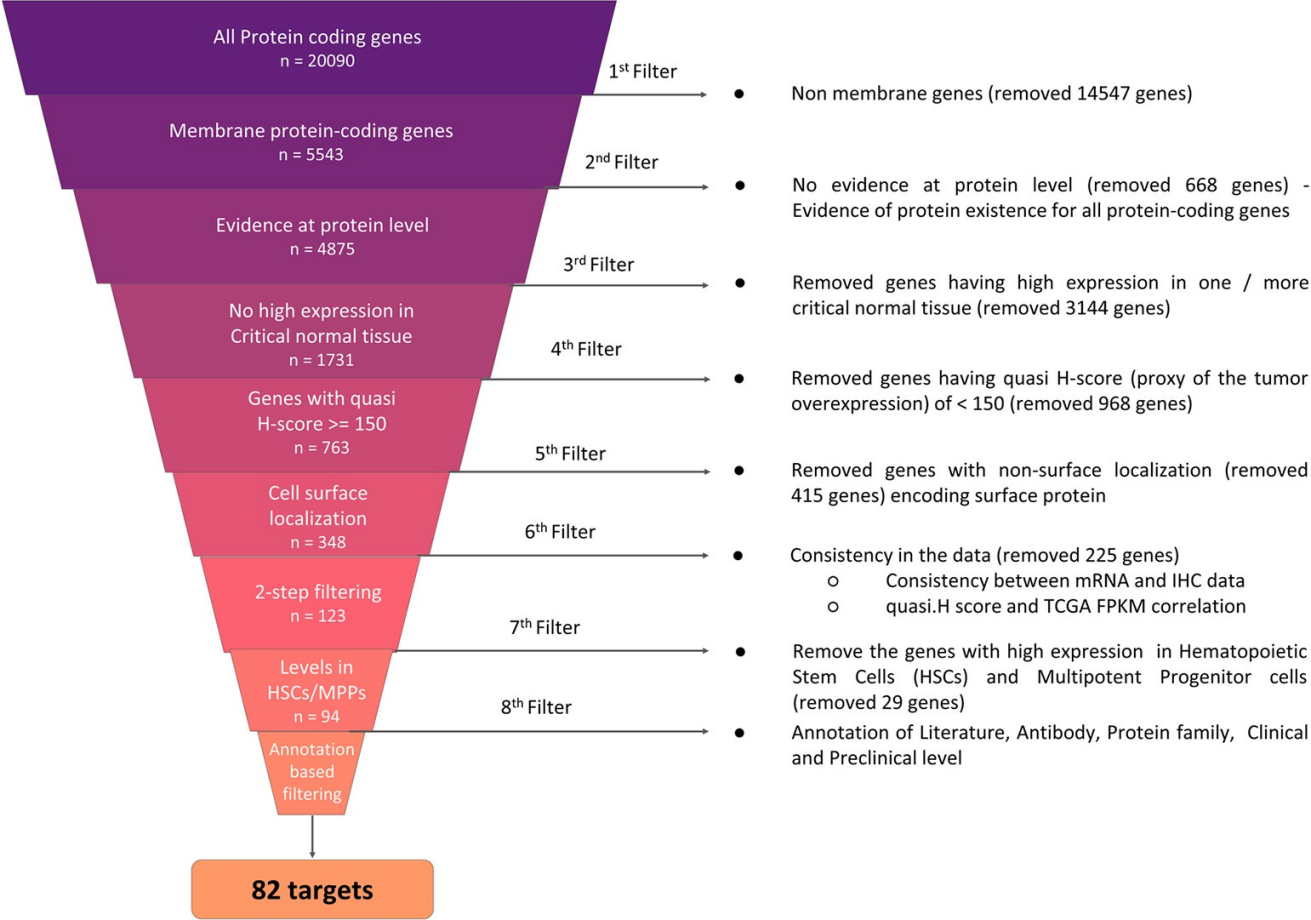
ADC target identification methodology workflow. Workflow depicting approach used for identification of 82 prioritized ADC targets starting from a list of 20K protein coding genes.

Derived from methods used by Razzaghdoust et al. [14] and delineated in the methods section, and in Fig 1, we initially identified 5543 membrane protein coding genes out of a total of 20,090 genes using HPA database version 22.0. For further analysis, 4875 genes based on evidence at protein level were retained. It is worth mentioning that the same gene, which has a membrane protein annotation, may also have the intracellular localization for it`s isoforms. This is seen for many clinically validated target antigens, such as CD276 and ERBB2, which carry two annotations-membrane protein and intracellular in the protein atlas database. Such antigens are retained in our approach. By relying on annotation used in the protein atlas database, we have exclusively filtered out proteins which lacked any membrane annotations for further evaluation.

In order to minimize possible side effects of ADC targeting on healthy cells, we considered the removal of genes with high expression levels in 13 critical normal tissues as used in [14]; lung, oral mucosa, esophagus, stomach, duodenum, small intestine, colon, rectum, liver, kidney, heart muscle, skin, bone marrow. This step resulted in 1731 genes for subsequent investigation. We prioritized potential targets exhibiting high expression levels on tumor cells; hence excluded any genes with low quasi H-score (<150) in any of the cancer types. Using this criteria, we retained 763 genes with a > 150 quasi H-score in at least one out of 20 tumor types. As a subsequent step, we filtered out genes which did not show cell surface localization using the annotation provided by in silico human surfaceome [16] publicly available database (http://wlab.ethz.ch/surfaceome).

Considering the diversity of data types, which included RNAseq, immunohistochemistry, HPA webportal data, calculated quasi H-score, we implemented two stringent filtering steps to identify potential ADC target candidates and excluded: 1) Any gene that didn’t exhibit consistency with both mRNA and IHC data for normal tissue and 2) any gene which did not show consistency with mRNA and calculated quasi H-score data for tumor types in TCGA.

Following this methodological filtration process, we derived a list of 123 genes out of which we considered 122 genes for further analysis, excluding one gene due to its absence in the GEO study (GSE42519) data [15].

Increased levels of the target expression on various blood cell types can limit the accumulation of ADCs at target tumor sites [8]. The lack of targeted antigens on hematopoietic stem cells (HSCs) provides an advantage, allowing normal blood cells to recover from HSCs following temporary depletion caused by ADCs [3]. Consequently, we eliminated the 28 targets that display high expression on blood cell types, such as HSCs and multipotent progenitor cells (MMPs), by using the data from GEO study GSE42519 [15]. This led to retention of 94 genes, which included 67 genes with medium and 27 with low expression levels on HSCs and MMPs, which is given in the S1 File.

**Fig 2:**
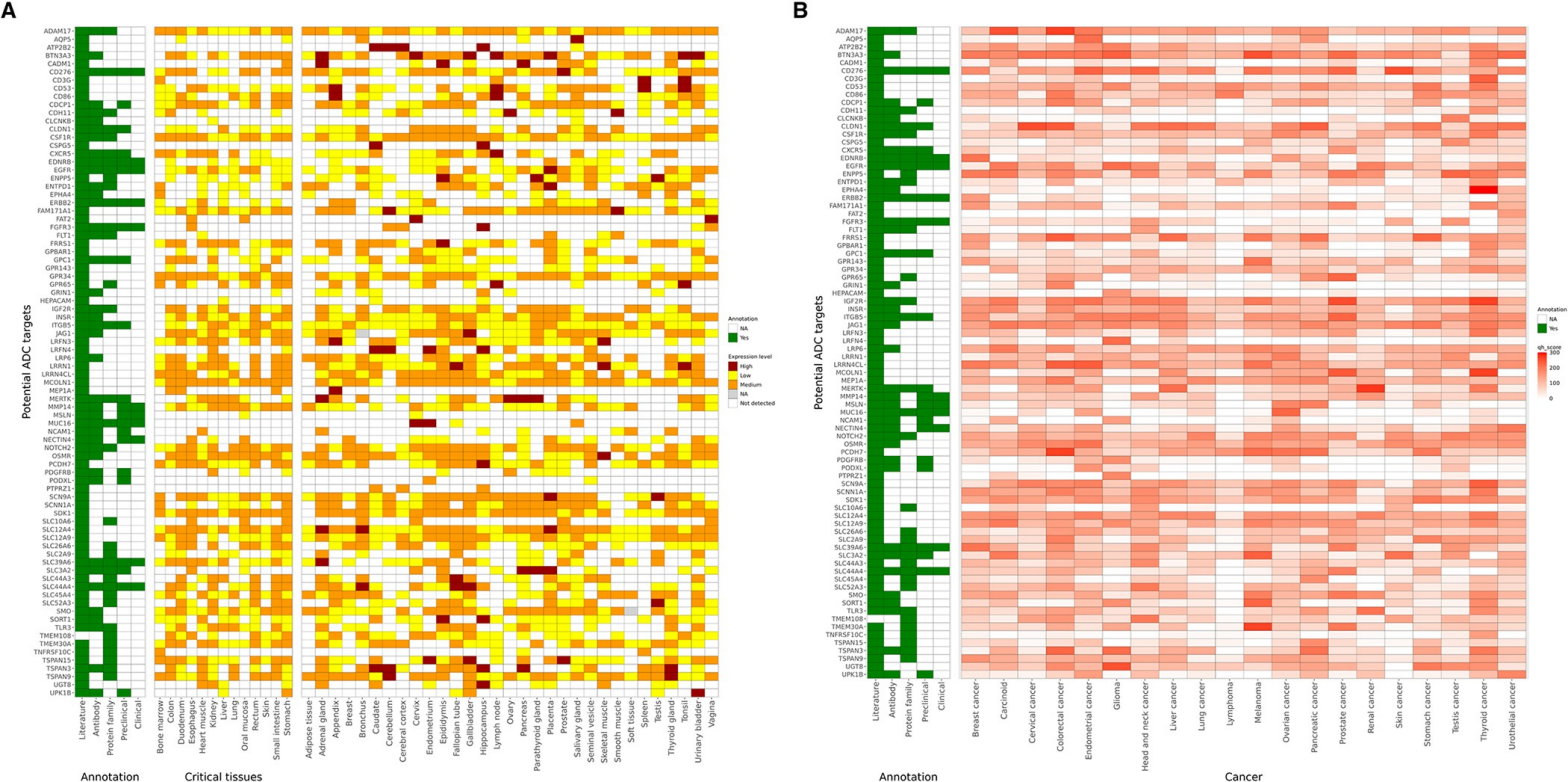
Expression of 82 prioritized ADC targets across normal and tumor tissues along with evidence based filtering annotations using five criterias*. **A)** A heatmap depicting expression levels of potential ADC targets across 44 normal tissue types. **B)** A heatmap depicting expression levels of potential ADC targets across 20 tumor types based on their quasi H score. *1) Literature: targets for which there is existing literature evidence elucidating their potential role in tumor biology. 2) Antibody: targets against which antibodies have been generated 3) Protein family: targets belong to a protein family where other proteins isoforms of which have been employed for the advancement of ADC in either clinical or preclinical setting 4) Preclinical: targets tested in preclinical setting 5) Clinical: targets tested in clinical setting.

In the final step, we applied five criteria to prioritize the targets and kept the ones which met at least one of these criteria. 1) Literature: targets for which there is existing literature evidence elucidating their potential role in tumor biology, 2) Antibody: targets against which antibodies have been generated, 3) Protein family targets: belong to a protein family, where other proteins isoforms within this family have been employed for the advancement of ADC in either clinical or preclinical setting, 4) Preclinical: targets tested in preclinical setting and 5) Clinical: targets tested in clinical setting. Total 82 prioritized targets navigated through the entire validation process are listed Fig 2A and 2B. Data of both figures are given in S2 and S3 Files.

40 out of these 82 prioritized targets show either no detection levels, low, or medium expression across all 44 normal tissues. 15 targets; AQP5, ATP2B2, CLCNKB, CSPG5, EDNRB, ENPP5, FLT1, GPBAR1, GRIN1, HEPACAM, MSLN MUC16, PODXL, PTPRZ1 and SLC2A9, exhibited low / not detected expression levels across all 13 critical normal tissues.

From our list of 82 prioritized targets, 22 have already been tested as ADCs in the preclinical or clinical settings, including HER2, NECTIN4 and EGFR, demonstrating the validity and potential of our approach.

We identified 60 additional targets which to our knowledge have not been used for ADC development. Our list included insulin-like growth factor-2 receptor (IGF2R) and SORT1, which have been explored for radioimmunoconjugate [18] and peptide-drug conjugate targeting, respectively [19]. The list included 19 targets against which antibodies have been generated in either oncology or non-oncology space, i.e, colony-stimulating factor-1 receptor CSF1R/CD115 against which monoclonal antibody emactuzumab is under clinical investigation [20]. The colony-stimulating factor 1 receptor (CSF-1R) functions as a transmembrane receptor tyrosine kinase, which is a receptor for colony-stimulating factor 1 (CSF-1) [21]. Intratumoral CSF-1/CSF-1R signaling has been reported to play a key role in triggering the recruitment of tumor-associated macrophages leading to tumor growth and facilitating metastasis [22–24].

Among the 60 remaining targets, as mentioned above, 22 belong to a protein family which has been employed for ADC development, i.e, One member of the ectonucleotide pyrophosphatase/phosphodiesterase 1 protein family, ENPP5, has been identified as a potential ADC target. Another protein from this family, ENPP3, underwent clinical trials for ADC development targeting renal cell carcinoma (RCC) [25]. Our analysis suggests that such targets may hold potential to be explored as ADC targets. An additional 28 out of these 60 targets or their protein families have not been explored for generation of ADCs or antibodies. However, there is existing literature evidence elucidating their potential role in tumor biology, i.e, UGT8 is one such target encoding a protein belonging to the UDP-galactose:ceramide galactosyltransferase family. UGT8 is an enzyme responsible for catalyzing the transfer of galactose molecules from UDP-galactose to ceramide, leading to the formation of galactosylceramide [26]. Elevated expression of UGT8 is reported in multiple malignancies such as breast, lung and prostate cancers [26–28].

Among our list of new potential ADC targets, there are a few intriguing candidates pertaining to a protein family that is being utilized as targets for ADC development, antibodies have been generated against them, and they have a well understood role in tumor biology. Examples include NOTCH2, against which monoclonal antibody tarextumab was generated [29], which has been tested in phase II clinical trials [30]. While an ADC against the protein family member NOTCH3 was subjected to clinical investigation [31] however, NOTCH2 has not been investigated as an ADC target. The biological significance of an ADC target is underscored by its overexpression in cancer cells, its key role in disease development, ability to facilitate ADC internalization, support from both preclinical and clinical research, and its restricted expression in normal tissues [32]. Further investigation is necessary to evaluate the internalization potential of these additional targets.

**Fig 3:**
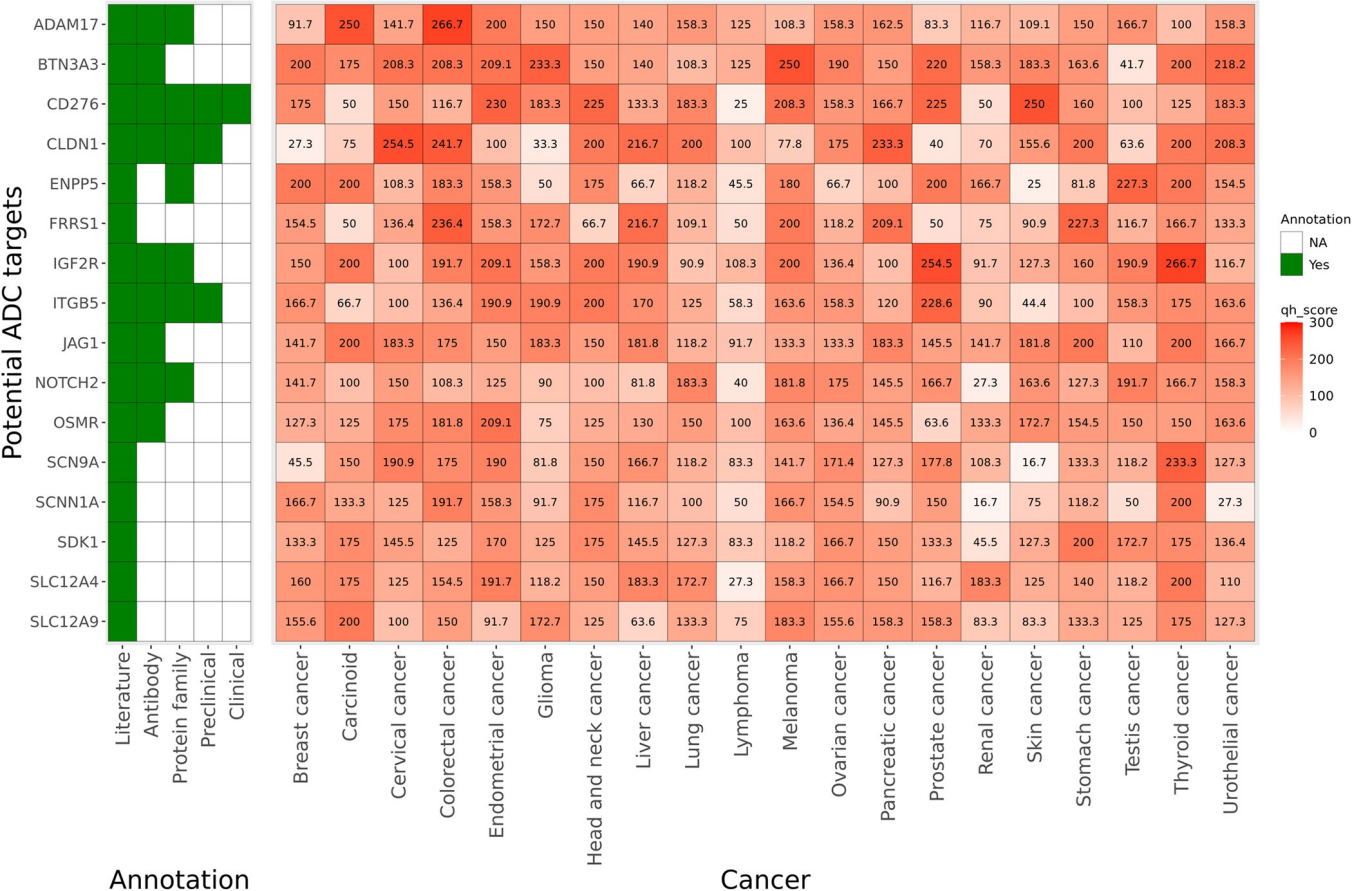
Expression levels of 16 targets with >150 quasi scores across more than 7 tumor types. A heatmap depicting quasi H-score across 20 tumor types for 16 potential ADC targets with >150 score in more than 7 tumor types.

We found 16 targets from our list were able to target more than 7 indications, with >150 quasi H-score (Fig 3), possessing substantial literature evidence indicating their potential role in tumor biology. This list includes CD276 or B7-H3 which is already under clinical investigation for ADC development. Another intriguing potential target candidate in this list is from non-oncology space, OSMR-receptor for Oncostatin M (OSMR), which exhibited overexpression across 10 cancer indications in our analysis. Fully human monoclonal antibody against OSMR has been generated and is in clinical trials for pruritus in prurigo nodularis [33]. Adequate preclinical data is present, substantiating that overexpression of OSMR results in unfavorable outcomes across a broad spectrum of tumor types [34–43].

**Fig 4:**
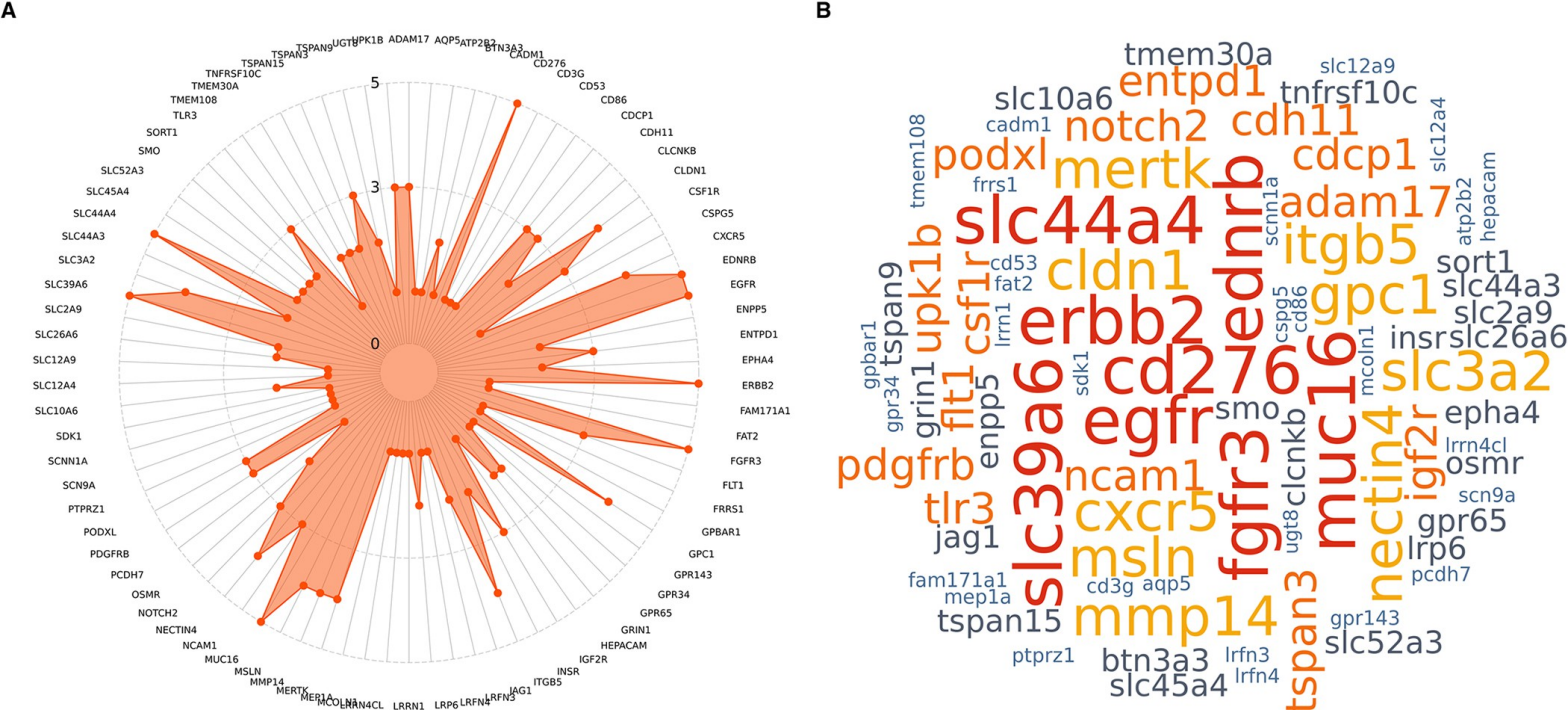
Scoring of 82 prioritized ADC targets based on five evidence based filtering criterias. **A)** Radar plot generated using five criterias mentioned in the method section to give scores between 1 to 5 in order to rank potential ADC targets. It shows 82 prioritized targets in a circular fashion and each point on the plot represents a corresponding score for the aligned target. **B)** A wordcloud representing potential ADC targets based on the five criteria annotations. Wordcloud is a representation of a score for each of the 82 prioritized targets by color and size of the word. The targets with the same score are represented by the same color and font size with 5 being the highest score and 1 being the lowest score.

Using the five criteria mentioned above, we generated a score between 1 to 5 (with 5 being highest) to rank potential targets which passed through in our screening. Fig 4A and 4B represent 82 targets along with their computed scores. We found 8 targets CD276, EDNRB, EGFR, ERBB2, FGFR3, MUC16, SLC39A6, and SLC44A4 with scores of 5 and 9 targets CLDN1, CXCR5, GPC1, ITGB5, MERTK, MMP14, MSLN, NECTIN4, and SLC3A2 with scores of 4.

It would be of interest to further evaluate the role of these targets in additional tumor indications, as well as their potential to serve as ADC targets.

### Exploring the impact of mutated genes on the expression levels of prioritized ADC targets

The process of payload internalization, retention and ADC efficacy is significantly influenced by target expression on the tumor tissue [44]. ADC targets under development often show heterogeneous expression profiles on tumor tissues [6]. A key aspect of tumor heterogeneity comes from genomic instability and the mutational landscape. Therefore, we employed TCGA mutation database to determine correlation between expression levels of targets and 416 mutated genes across 22 tumor types for 82 prioritized targets. We found that 336 out of 416 mutated query genes significantly altered the expression of 46 out of 82 targets. To identify a strong correlation, we exclusively considered targets showing a log2 fold change greater than or equal to 1, in conjunction with the cancer subtype exhibiting a population change of 5% or more due to the specified mutation. Our analysis showed that the KRAS mutation altered the expression of 23 targets across 4 tumor subtypes, while the p53 mutation affected the expression of 16 targets across 10 tumor subtypes. TCGA tumor type abbreviations are given in S4 File.

**Fig 5:**
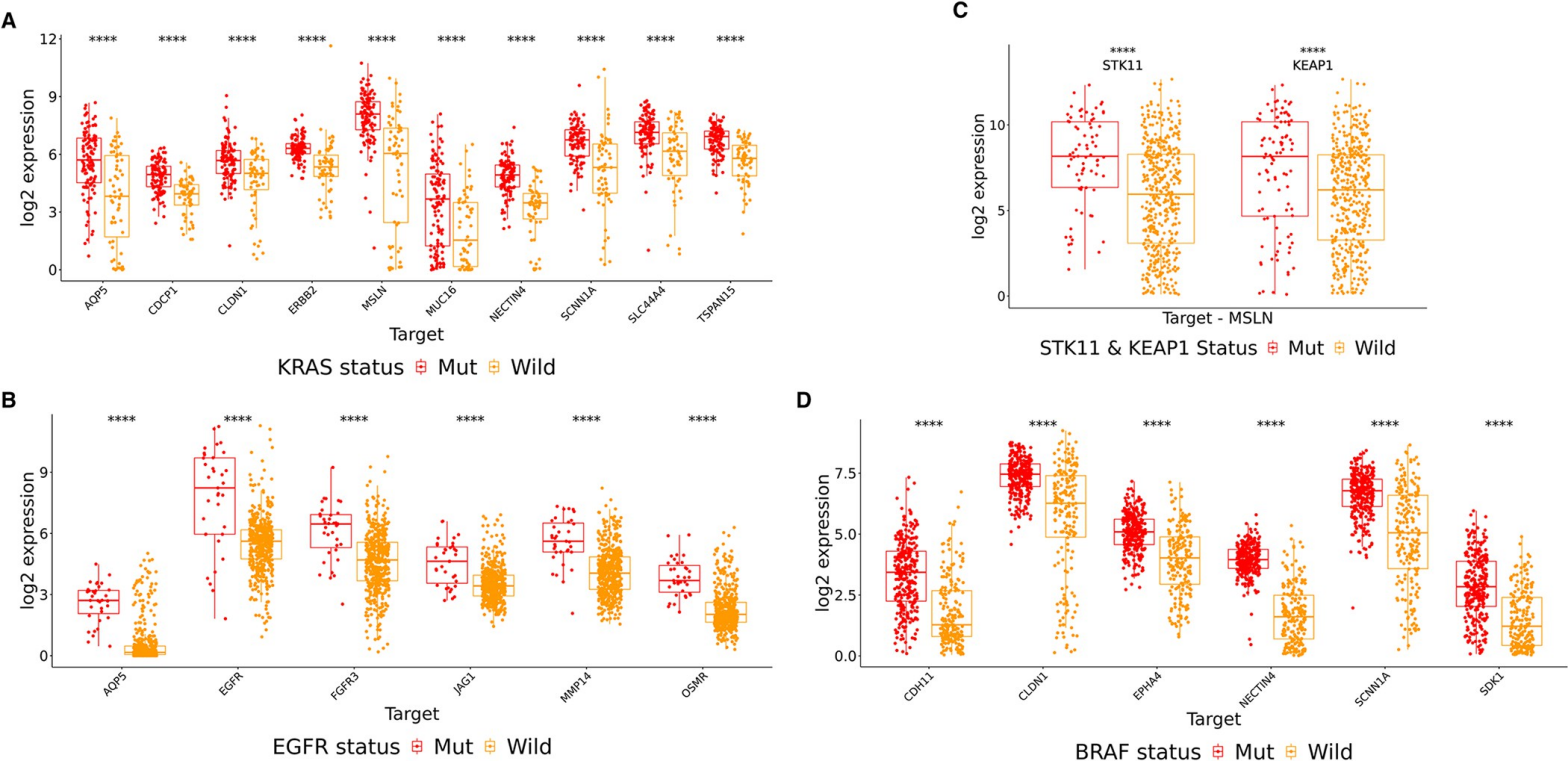
Impact of mutations on expression levels of ADC targets identified in our analysis across tumor subtypes. **A)** Impact of KRAS mutation on expression levels of multiple targets in Pancreatic Adenocarcinoma (PAAD) **B)** Impact of EGFR mutation on expression levels of multiple targets in Low Grade Glioma (LGG) **C)** Impact of STK11 and KEAP1 mutation on expression level of MSLN in Lung Adenocarcinoma (LUAD) **D)** Impact of BRAF mutation on multiple targets in Thyroid Carcinoma (THCA). The annotations are given as “Mut” for mutated gene and “Wild” for wild type gene.

RAS, comprising 3 genes, H-RAS, K-RAS and N-RAS, that encode proteins that play critical roles in key cell signaling pathways, and is the second most prevalent gene driver mutation across diverse human cancers, manifesting in 20% to 30% of all human malignancies [45]. Notably, K-RAS is the most frequently mutated of the three RAS genes, with the oncogenic variant being detected in approximately 88% of pancreatic cancer cases [46]. The results of our mutation analysis revealed upregulation of 10 targets AQP5, CDCP1, CLDN1, ERBB2, MSLN, MUC16, NECTIN4, SCNN1A, SLC44A4, and TSPAN15 in KRAS mutated pancreatic adenocarcinoma (PAAD), unlocking their potential to provide clinical benefit in this subset of patient population (Fig 5A).

Recent investigations report an elevated occurrence of EGFR mutations, in low-grade gliomas (LGGs) reaching up to 23% [47]. EGFR-mutated LGGs display a poorer overall survival outcome [48]. Our analysis revealed that alteration in the EGFR gene can lead to upregulation of 2 clinically tested ADC targets, FGFR3 and MMP-14, and one new potential target OSMR in LGGs (Fig 5B). Developing ADCs using targets overexpressed in EGFR-mutated LGGs holds the potential clinical advantages.

MSLN showed a 4.37 and 2.61 absolute fold upregulation in STK11 and KEAP1 mutated lung adenocarcinoma (LUAD) patient population, respectively (Fig 5C). Our analysis suggests that ADCs targeting MSLN may be particularly beneficial in lung cancer patients harboring dual mutations in STK11 and KEAP1 genes.

We observed that BRAF mutations led to change in the expression level of 7 targets most prominently in thyroid carcinoma (THCA) (Fig 5D). This included upregulation of NECTIN4, a target used in the approved ADC, Enfortumab Vedotin. Another study group reported that more than 50% of patients with THCA had BRAF mutant samples [49] which might provide a possible explanation for our observations.

It’s important to highlight that mutations in a gene can exert varied impact on the target expression level, depending on the tumor type. For example, our analysis highlighted that mutation in tumor suppressor protein p53 coding gene TP53, correlates with the upregulation of MSLN in breast invasive carcinoma (BRCA) and PAAD. Conversely, it correlates with the downregulation of MSLN in cervical squamous cell carcinoma and endocervical adenocarcinoma (CESC). Additionally, we observed TP53 mutation in BRCA correlates with 4.50 fold downregulation in SLC39A6/LIV-1 expression, corroborating with the results published by fang et al [8].

**Fig 6:**
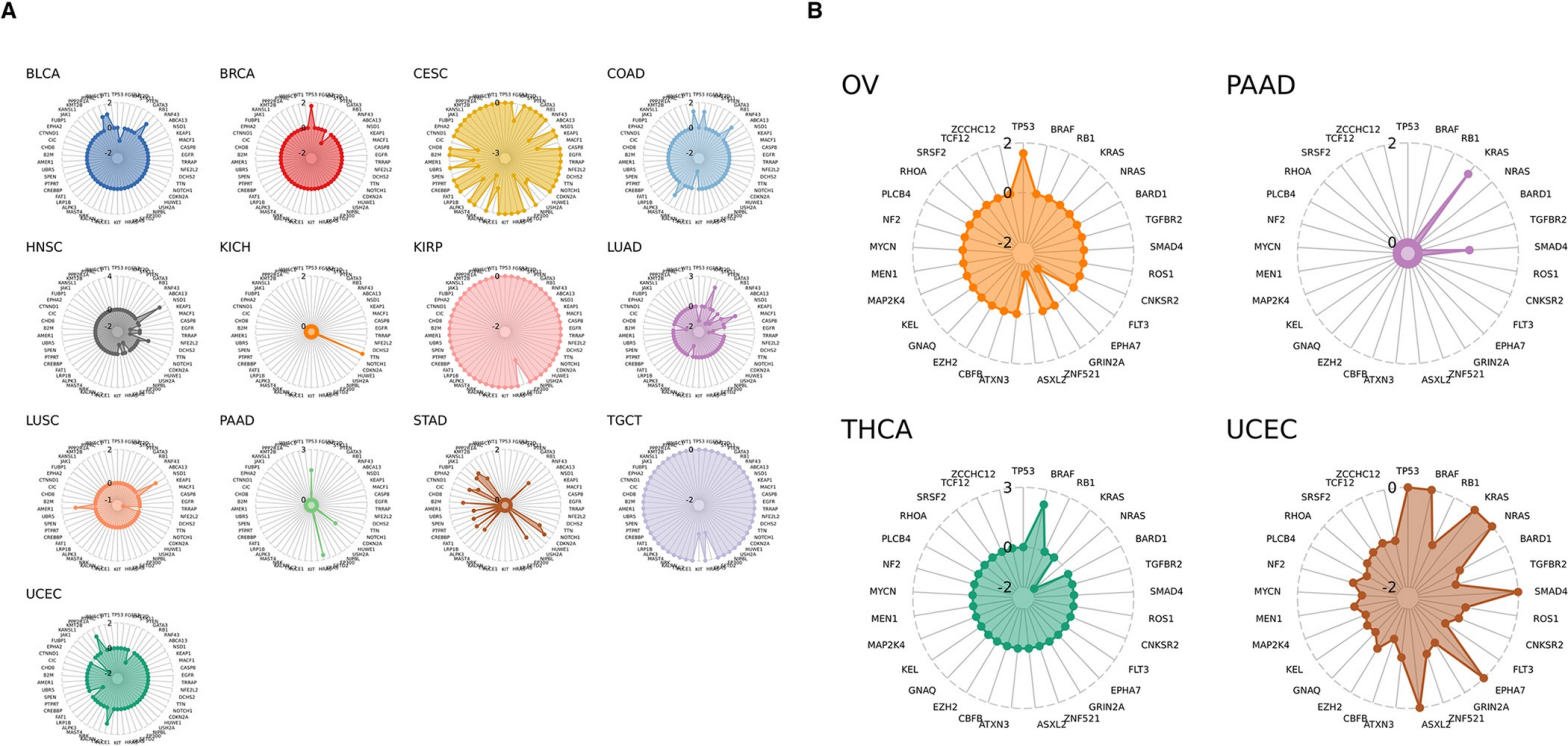
Mutations impacting MSLN and NECTIN4 target expression level across tumor subtypes. **A)** Radar plot shows log2 fold change of MSLN target expression level across multiple tumor subtypes and mutations. **B)** Radar plot shows log2 fold change of NECTIN4 target expression level across multiple tumor subtypes and mutations.

Similarly, a single target expression can significantly vary depending on the combination of tumor type and gene mutation. For example, expression of MSLN was upregulated in 35-tumor type gene mutation combinations, while it was downregulated in another 33-tumor type gene mutation combinations as shown in Fig 6A. Another insight which can be extracted from our analysis is related to FDA approved ADC target NECTIN4, which was upregulated in 4 tumor type gene mutation combinations including OV/TP53, THCA/BRAF, PAAD/KRAS, PAAD/SMAD4, and was downregulated in 25 tumor type gene mutation combination. 21 of these mutations resulted in the downregulation of the NECTIN4 expression, specifically in the Uterine Corpus Endometrial Carcinoma (UCEC), as illustrated in Fig 6B.

Tumor heterogeneity can impact ADC target expression leading to uneven binding and reduced efficacy. This may result in resistant tumor subpopulations, limiting the overall therapeutic response [50]. Understanding the impact of mutations on heterogeneous target expression patterns in cancers can help improve treatment response and provide an approach for further personalized oncology using ADCs.

### Identification of potent tumor selective payload candidates

**Fig 7:**
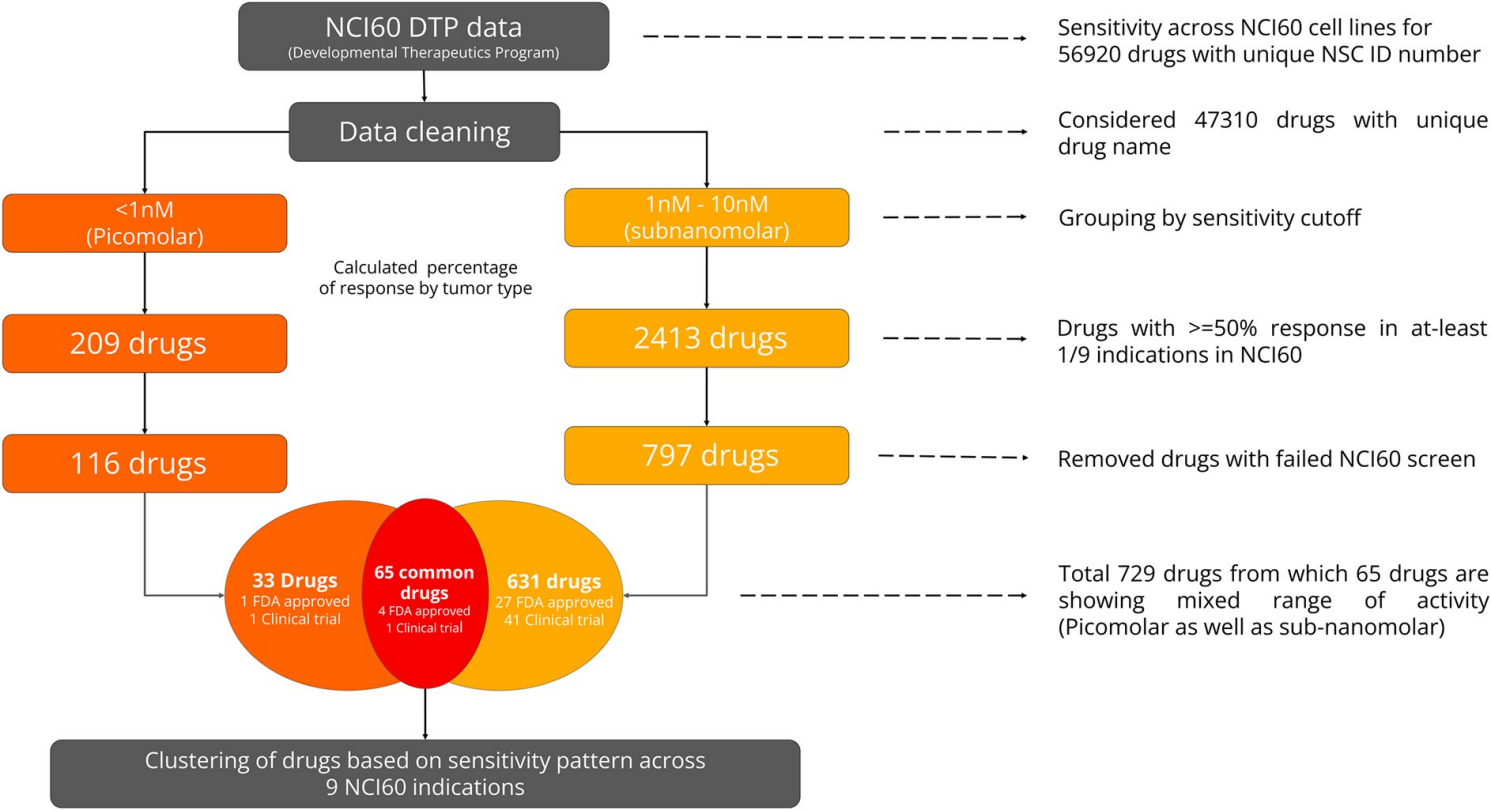
Workflow used to identify potent payload candidates in a tumor selective manner. Workflow depicting strategy employed to identify tumor selective potential payload narrowing down from ~50K to 729 compounds.

We analyzed more than 50K compound data from the NCI-DTP portal. Following procedures outlined in the methodology section and shown in Fig 7, we categorized 47,310 unique compounds based on their sensitivity level into two groups: a) compounds exhibiting picomolar (<1nM) range and b) compounds exhibiting low nanomolar (1nM - 10nM) range sensitivity. Subsequently, compounds that have > = 50% response in at-least 1/9 indications in NCI60 were retained, leading to a total of 209 compounds in the picomolar group and 2413 compounds in the low nanomolar group. In the next step, compounds which failed NCI60 screening were eliminated, which led to the removal of 93 and 1616 compounds from picomolar and low nanomolar groups, respectively. This resulted in a total of 729 compounds. 33 compounds grouped in the picomolar group, 631 compounds in the low nanomolar group and 65 compounds exhibited activity in both picomolar as well as low nanomolar range across NCI60 9 cancer indications.

In the resulting picomolar group, 1 compound is FDA approved and 1 reached clinical trial stage, while in the low nanomolar group, 27 compounds are FDA approved and 41 reached clinical trial based on NCI60 annotations. Among the compounds common between both subgroups, there are 4 FDA-approved and 1 compound which reached clinical trials. Using a hierarchical clustering method, in order to identify similar or contrasting sensitivity patterns, we subdivided the 33 compounds from the picomolar group into 5 clusters. 631 compounds from low nanomolar range and 65 overlapping compounds from both sensitivity groups (picomolar and low nanomolar) were subdivided into 10 clusters each.

### Screening of potential payloads displaying picomolar to ≦ 1nM sensitivity (using ≦ 1nM as cutoff)

**Fig 8:**
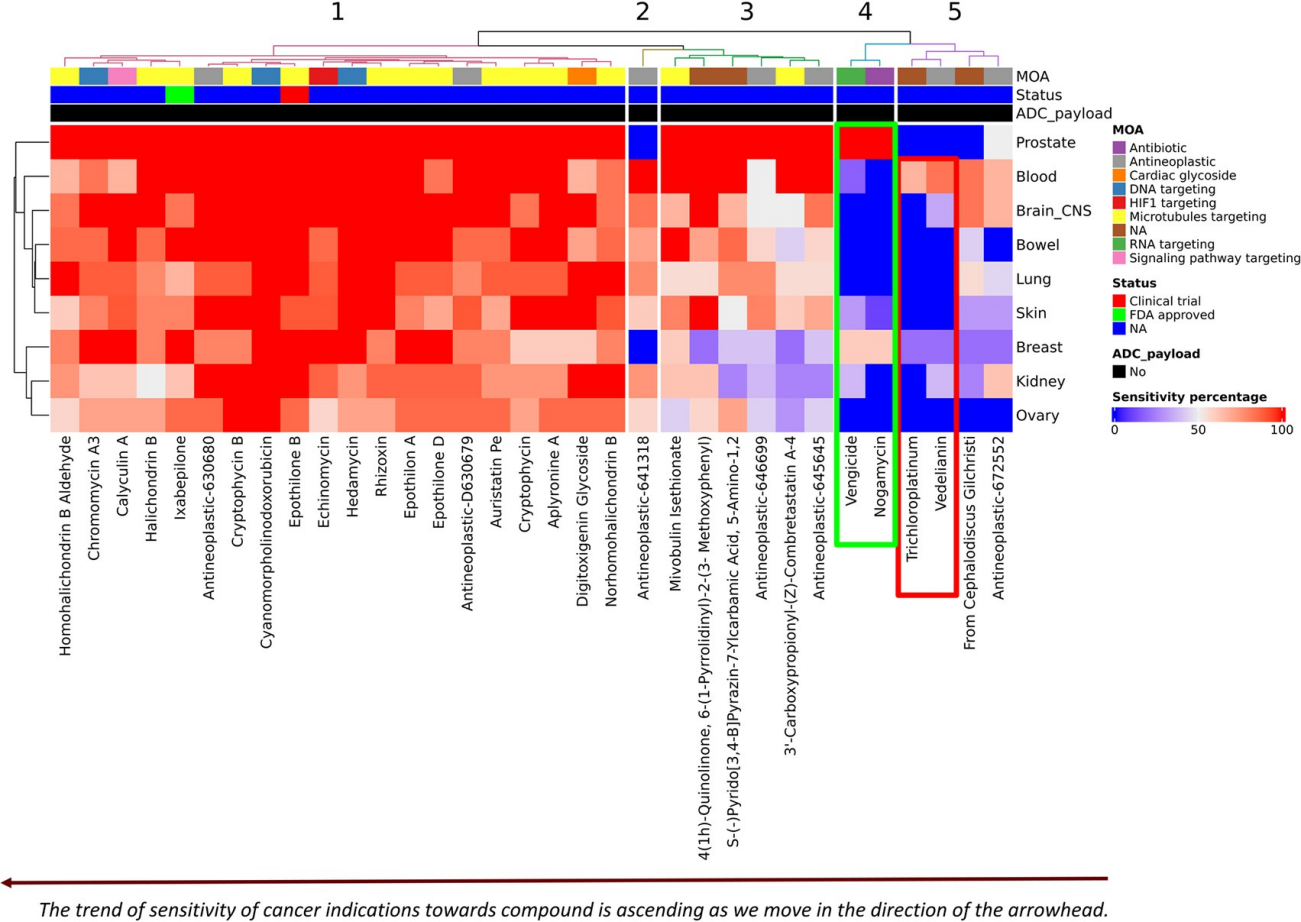
Heatmap depicting compounds with sensitivity ranging between pM to 1nM. Heatmap depicting clustering of 33 compounds based on sensitivity patterns across 9 NCI60 cancer indications. This figure represents a narrowed-down list of compounds that demonstrate specific or heightened sensitivity towards the desired cancer type. The trend of sensitivity of cancer indications towards compounds is ascending as we move in the direction of the arrowhead. It is feasible to identify compounds that exhibit distinct activity either in solid tumors or hematological malignancies, as shown in green box compounds, such as Nogamycin and Vengicide exhibit activity against prostate and breast cancer, while no activity was seen against heme malignancies at this sensitivity range. Red box highlights compounds, such as Vedelianin and Trichloroplatinum which exhibit differential activity, with blood cancers showing greater sensitivity.

Fig 8 is a representative heatmap of the 33 compounds with sensitivity ranging between pM to 1nM. The trend of sensitivity of cancer indications towards compounds is ascending as we move in the direction of the arrowhead. The majority of the compounds showing picomolar potency towards all 9 cancer indications are microtubule targeting agents isolated or derived from marine organisms, i.e.,apyroline, epothilone, halichondrin and cryptophycins. A large number of ADCs in clinical development use maytansinoid derivatives as payload, which is a substrate of multidrug resistance protein 1 (MDR1) limiting the clinical efficacy of ADCs due to emergence of drug resistance [51]. This drug resistance is predominantly caused by increased expression of multidrug transporters, like P-glycoprotein (MDR1/ABCB1) [52].

Therefore, it becomes imperative to identify potential payloads which can elude multidrug resistance (MDR) mechanisms. Cryptophycins are one of these potential payloads which are active against MDR cancer cell lines [53]. It failed to show single- agent efficacy in clinical trials but has re-attracted interest as a promising ADC payload [51]. Another compound identified from our compilation is a colchicine analog, mivobulin isethionate, which is also a microtubule targeting agent, that demonstrated broad range antitumor activity in cell lines exhibiting MDR in preclinical evaluation [54]. It failed to show efficacy as a single-agent in earlier clinical trials [54–56]. However, it may be of interest to explore the possibility to repurpose such compounds or their analogs as ADC payloads in a tumor selective manner.

By employing our strategy, it becomes feasible to identify compounds that exhibit distinct activity either in solid tumors or hematological malignancies. For example, our clustering results found nogamycin, an anthracycline, to show limited activity in hematological cancer cell lines at the picomolar level, while it showed 100% activity in prostate cancer, followed by 60% activity in breast cancer cell lines. Similarly, our compilation indicated that vedelianin exhibits differential activity, with blood cancers showing greater sensitivity. A recent review emphasized the potential of exploring Golgi apparatus targeting compounds to create innovative therapeutic agents against cancer cells [57]. Vedelianin could potentially hold intriguing characteristics due to its disruptive effects on Golgi apparatus [58]. It has been reported to show antiproliferative activity at low nanomolar concentrations in tumors. Notably a path for a fully synthetic process for this molecule has been published [59].

### Screening of potential payloads with (low nanomolar) 1nM to ≦ 10nM sensitivity (using ≦ 10nM as cutoff)

S1 File is a representative heatmap of the 631 compounds with sensitivity ranging between 1nM to 10nM. This group of compounds include many FDA approved ADC payloads such as Dxd, exatecan mesylate and MMAE. The complete table of 631 compounds along with their annotations is provided in S5 File. By implementing the approach of using GI_50_ and sensitivity pattern across 9 different cancer indications, we were able to cluster together compounds which share sensitivity profile with successful ADC payloads and can be explored as potential new payloads. One such compound is, N-[[(1R,2S,10R,12R,13S)-12-Cyano-8,19-dihydroxy-5,7,16,18-tetramethoxy -6,17,21-trimethyl-11,21-diazapentacyclo[11.7.1.02,11.04,9.015,20]henicosa-4(9),5,7,15(20),16,18-hexaen-10-yl]methyl]quinoxaline-2, which exhibits a comparable sensitivity profile to maytansinoid derivatives. These compounds could be considered an intriguing candidates for more extensive assessment as possible novel payloads.

Our analysis may help identify novel potential payloads with diverse mechanisms of action and selective tumor targeting. One of the compounds identified in our screening are Illudins, a class of natural compounds, derived from Jack-o’- Lantern Mushrooms [60]. Illudins have demonstrated antitumor efficacy at nanomolar levels and have already been explored as potential payload using docking simulation by other reports [61]. Illudin derivatives may offer selective targeting due to reliance on enzyme Prostaglandin Reductase 1 (PTGR1) to the desired tumor types, which can lead to optimal results by controlling off-target toxicity in ADCs [62].

It may be possible to select suitable payloads to pair with tumor types, such as kidney and ovarian cancers, which have shown maximum variability in their sensitivity pattern towards listed compounds in this subclass. We found mTOR and dual PI3K/mTOR inhibitors, such as sapanisertib, everolimus and omipalisib, to show significant and increased specific activity against kidney cancer cell lines, which is consistent with other reports [63]. This emphasizes the significance of employing payloads, which can effectively target mTOR signaling pathways, in designing ADC targeting strategies against kidney cancer. Similarly, another compound identified in our screening is BRD-K58304294-001-01-5, which is a potential piperidine derivative which exhibited specific activity against ovarian cancer cell lines in the 1nM to ≦ 10nM sensitivity range.

It is worth understanding that effectiveness of ADCs may be influenced by the physicochemical characteristics of payloads. For example, MMAF, with limited cell permeability, relies on high tumor antigen expression for efficacy but lacks bystander killing [64]. On the other hand, as a free drug, MMAE is more potent than MMAF due to its increased cell permeability, allowing it to diffuse out of the target cell and cause bystander killing in surrounding cells [65, 66]. This distinction emphasizes the trade-offs between cell-specific targeting and broader cytotoxicity in the design and effectiveness of ADCs. The novel payloads identified in our screening method deserve additional evaluation to determine their chemical characteristics and suitability for conjugation.

### Screening of potential payloads with overlapping sensitivity in picomolar to low nanomolar (using ≦ 10nM as cutoff)

**Fig 9:**
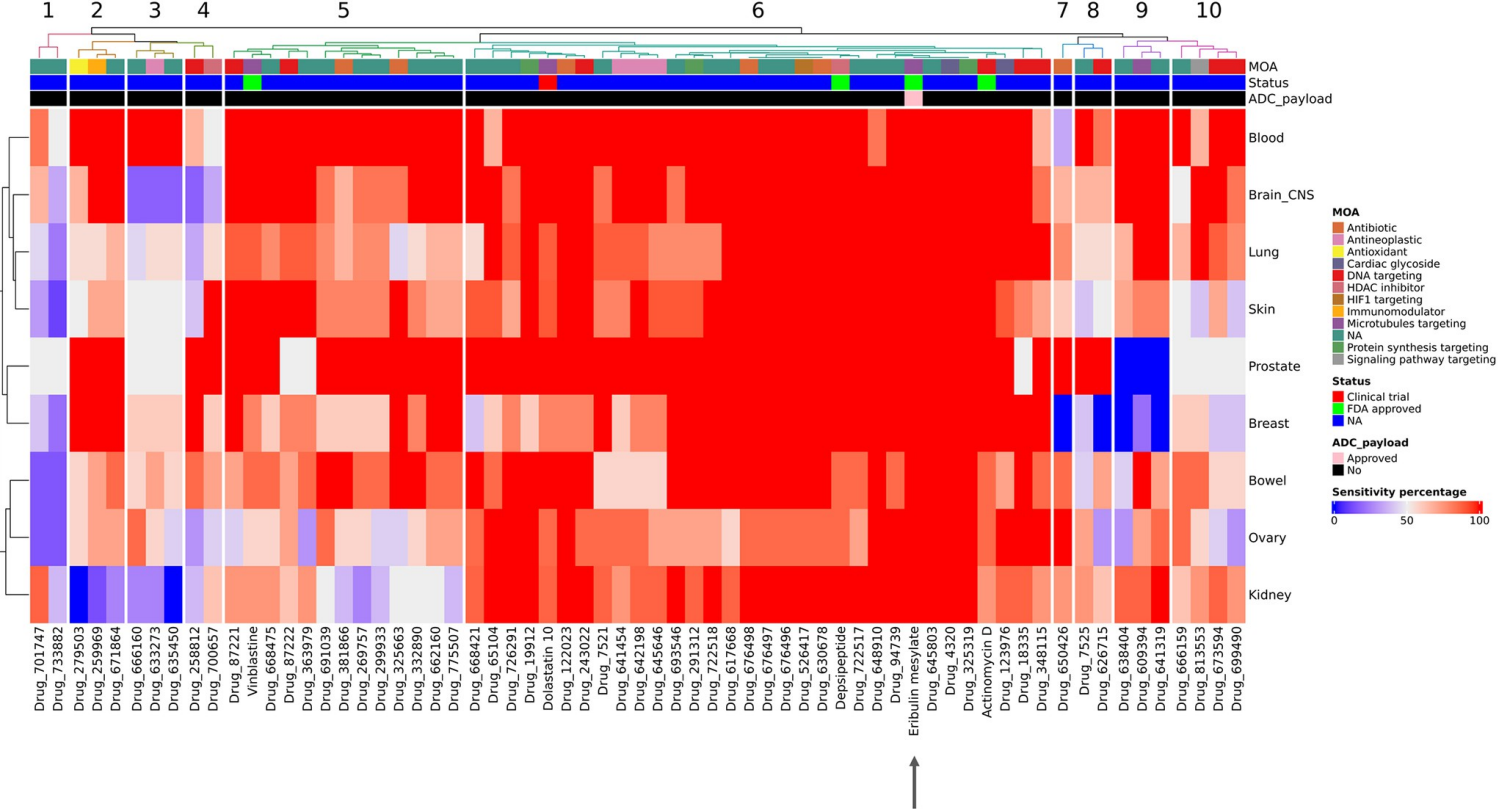
Heatmap depicting compounds with sensitivity ranging between picomolar to ≦ 10nM. Heatmap depicting clustering of 65 compounds (overlap with both subclasses) based on sensitivity patterns across 9 NCI60 cancer indications. Arrowhead represents eribulin mesylate, an ADC payload identified in this subclass which is currently under active clinical investigation.

Fig 9 is a representative heatmap of the 65 compounds showing overlap with both of the subclasses with broad sensitivity ranging between picomolar to ≦ 10nM. Complete table of these 65 compounds is provided in the S6 File. This group included eribulin mesylate, which is under active clinical investigation as an ADC payload [67].

**Fig 10:**
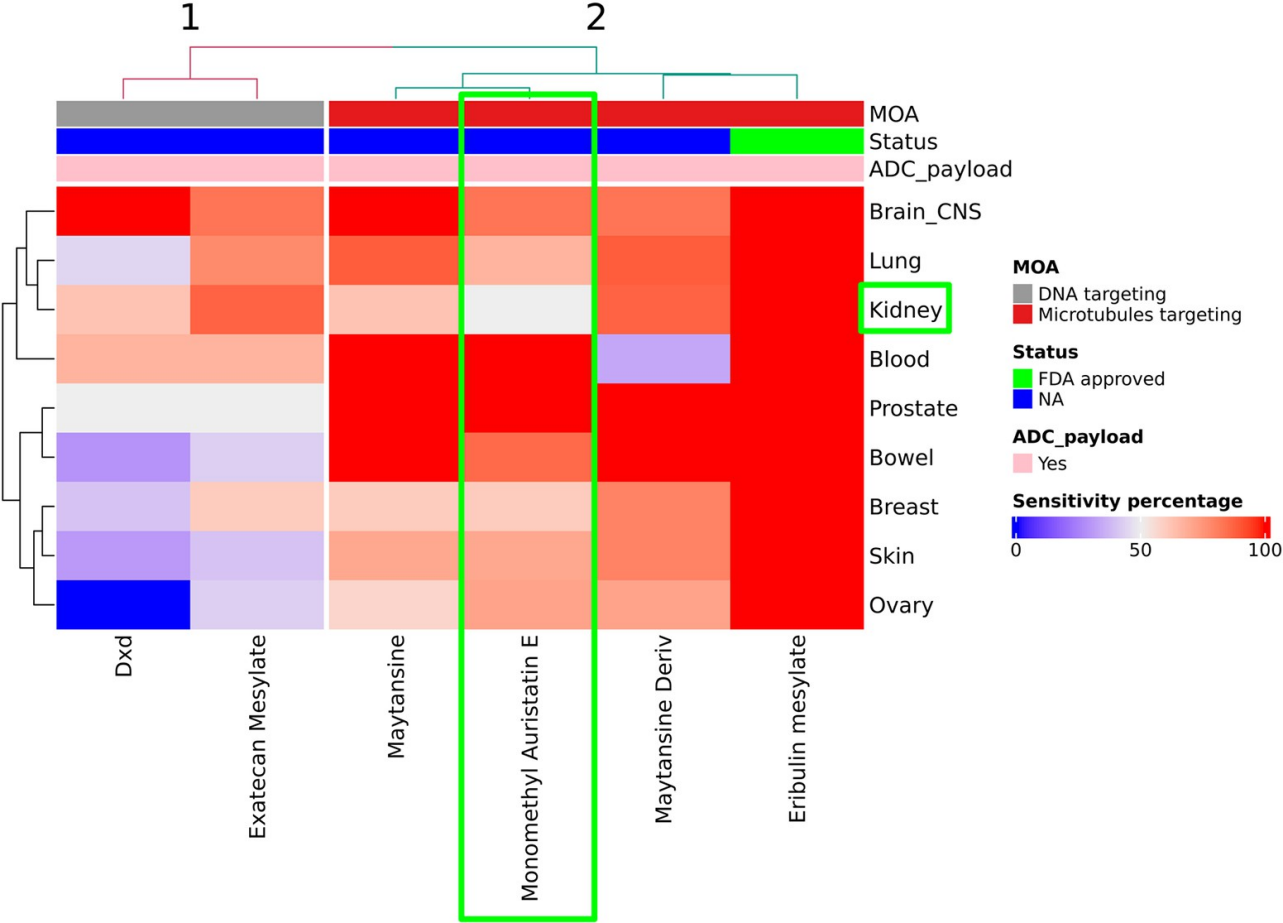
Heatmap representing clinically tested ADC payload. Heatmap depicting clustering of 6 compounds identified in our screening based on sensitivity patterns across 9 NCI60 cancer indications. As highlighted by green boxes utilization of MMAE, expressed moderate activity against renal cell carcinoma, a discernible pattern reported by other studies, which aligns with our analysis [68].

Fig 10 shows a heatmap of ADC payloads, which are under clinical development and exhibit sensitivity in picomolar to **≦**10nM (using **≦**10nM as cutoff) range. As an illustration, our clustering analysis revealed that MMAE exhibited only moderate activity in the context of renal cancer cell lines, which aligns with another study highlighting the intratumoral disposition of MMAE can potentially contribute to its moderate activity in RCC [68]. It is worth noting that many ADCs inactivated/discontinued for RCC were using maytansinoid/MMAE derivatives. Although an interplay of all three key components of ADCs and tumor specific characteristics might have contributed to the deactivation of these assets for RCC positioning, a discernible pattern aligns with our analysis.

### Identification of target-indication-payload combination

The effective design of an ADC requires that the target antigen and corresponding payload work synergistically. We aligned 9 tumor indications from NCI60 with the target antigen and selected clinically tested payloads Dxd, exatecan mesylate, maytansine, monomethyl auristatin E, maytansine derivative, eribulin mesylate as described in methods section. Subsequently, we incorporated mutation data with the target-indication-payload combinations. The corresponding data is provided in the S7 File.

Potential of ADC for glioma patients lacks clarity. Previous attempts using auristatin based payload did not enhance overall survival in newly diagnosed glioblastoma as a monotherapy [69] while AMG-595 employing maytansine based payload DM1 showed promise in glioma [70]. Our approach identified eribulin mesylate, DXd and maytansine as suitable payloads for pairing with target antigen EGFR, while excluding auristatin based payloads. Preclinical investigations validate the capability of eribulin to penetrate brain tumor tissue [71] and is reported to demonstrate efficacy in controlling brain metastasis in breast cancer [72]. Similarly, results from the phase II trial HERTHENA-Lung01, demonstrated a 33.3% central nervous system (CNS) response rate in patients with brain metastases treated with ADC patritumab deruxtecan, which utilizes Dxd as a payload [73]. These validation strengthens our study methodologies, providing valuable insights for future research in the field.

Patients with STK11/KEAP1-mutant lung adenocarcinoma may experience limited benefit from checkpoint blockade therapies highlighting unmet need for improved treatment strategies [74, 75]. Our analysis suggests that designing a MSLN-directed ADC carrying eribulin mesylate as a payload may be beneficial for STK11/KEAP1-mutant lung adenocarcinoma patients. It’s worth understanding that these insights need further investigation of target, linker and payload combination selection, along with considerations of stage and characteristics of tumor specific biology.

## Discussion

Through our thorough analysis, we pinpointed a set of 82 prioritized ADC targets and 290 target indication combinations for precise targeting of tumors. Among these, 22 ADC targets have already undergone evaluation in clinical trials or preclinical contexts, including ERBB2 and NECTIN4 demonstrating the validity of our approach. We have identified 60 additional novel targets that meet our filtering criteria and have not yet been investigated for ADC development. One of the novel targets identified by our approach is OSMR-receptor for Oncostatin M (OSMR), which exhibited overexpression across 10 cancer indications. OSMR is a member of the GP130 cytokine receptor family, which upon OSM ligand binding can lead to activation of signaling pathways such as the JAK/STAT, MAPK, and PI3K/AKT [34]. Fully human mAb that blocks OSMR beta are in clinical trials for pruritus in prurigo nodularis [33]. Despite of ample preclinical data available about OSMR `s association with poor outcome in cancers including ovarian, synovial sarcoma, pancreatic, gastric, glioblastoma, breast, cervical and bladder cancer [34–43], its clinical exploration within the field of oncology has not yet taken place. These targets could hold potential for application in the development of ADCs targeting [35] cancers. Our results suggesting modulation in target expression based on mutational profile of tumors emphasize that selection of ADCs should not solely be determined by the tumor type, but should also consider the specific genomic profile of these tumors. Knowing that specific tumor mutations can impact target expression can be valuable in early clinical trials, correlating with response depth. As additional ADC treatment options emerge, such data may eventually aid in selecting the most effective ADC based on the genomic mutational context of the tumor.

We acknowledge that the disposition of ADCs can be influenced by a multitude of factors beyond the scope covered in our work. The optimization of ADC design includes ensuring efficient internalization rates and gaining understanding of the mechanisms of elimination [4, 76, 77]. Future ADC design may incorporate strategies to further enhance therapeutic efficacy and minimize off-target effects. The present study has certain limitations. We opted for HPA datasets because they offer data from IHC, which can be more accurate than mRNA expression data. However, there are limitations due to low sample sizes of IHC data for each cancer type. To ensure robust potential results, we focused solely on common target antigen with high expression in both TCGA mRNA data as well as IHC datasets. Our selection was guided by the Surfaceome list provided by the literature [16]. Moving forward, we intend to investigate additional databases to further validate our findings. Some of the targets were omitted during our screening process, examples include TROP2, HER3 and CLDN18.2. Potential reasons for this could involve: (1) Utilization of a high quasi H score considering 150 as cutoff (ranging from 0–300) which eliminates several targets, (2) Our selection ensures that none of the resulting targets are highly expressed in 13 normal critical tissues to minimize toxicity and (3) In certain cases IHC data was missing from HPA dataset and we computed target levels using corresponding mRNA expression levels. While our analysis does not cover the gene fusions and additional omics data, such as copy number variation, it is comprehensive and covers a range of gene alterations, including point mutations, frameshift mutations, deletions and splice site mutations. One of the targets identified is a type I transmembrane protein, PODXL, which is reported to be expressed by kidneys, hematopoietic and vascular cells [78]. However, our database did not mark this as one with high expression in any of the critical tissues. It is worth mentioning that expression data are relative and the expression level marked as not detected represents the lowest relative expression. PODXL showed upregulation in endometrial cancer in our analysis. Another study reported the generation of a monoclonal antibody against PODO447, predominantly binding to a glycoepitope on PODXL. PODO447 not only exhibited specificity against PODXL tumor cell lines, but also demonstrated no reactivity against normal primary human tissues, including PODXL kidney podocytes. Notably, ADC based on PODO447 demonstrated specific efficacy *in vitro* for killing tumor cells [78] indicating its potential to be used for ADC target development.

Our payload mining approach serves as a valuable starting point, presenting a compilation of compounds exhibiting tumor selective responsiveness for use as potential ADCs payloads in precision medicine approach. It’s worth highlighting that many highly potent cytotoxic agents or their analogs were previously set aside, primarily those obstructed by toxicity constraints directly as sole therapeutic agents. The avenue of ADCs holds promise as a means to salvage these agents, valuable as payloads, due to their intrinsic attributes such as elevated cytotoxicity and mode of action [51]. Our compilation could contribute to the repurposing of existing cytotoxic agents, such as cryptophycins and illudins, to expand the arsenal of ADC-payloads in a tumor selective manner.

Some of the limitations associated with our payload mining strategy are as follows: (1) Potency of free cytotoxic agents is not the sole determinant for its suitability as ADC payload and our current work does not consider physicochemical characteristics of payloads [4], (2) We exclusively focused on compounds displaying GI_50_ up to 10nM. However, there’s a possibility that certain compounds slightly surpassed the 10nM cutoff and were excluded from our analysis, (3) We employed a 50% cutoff to retain compounds demonstrating a minimum of 50% activity in 1/9 cancer indications. The outcomes could differ based on the variation in cutoffs, (4) While examining a specific compound, there could be instances where it exhibits considerable sensitivity in certain indications; however, our analysis might reveal a comparatively lower sensitivity. For instance our analysis indicated a diminished level of sensitivity of Dxd against breast cancer, whereas Dxd is a clinically approved ADC payload against breast cancer. NCI-DTP covers 5 breast cancer cell line data and in our selected range Dxd shows sensitivity in 2 out of 5 cell lines, while 3 of those are falling outside our cutoff. Understanding the genetic and mutational profile can help uncover further specificity of these payloads, (5) Another limitation is posed by the availability of fewer cell lines. For example in case of prostate cancer there is availability of data for only two cell lines in the NCI-DTP data, making it difficult to draw definitive conclusions.

Constraints, such as a small sample size and limited indications, can be addressed by using large datasets like CCLE and GDSC covering ~300 drugs, >1,000 cell lines and >20 indications. By employing a strategy to use additional datasets it will be possible to generate more information regarding the genomic context of payload response, which will further refine the selective payload targeting. Furthermore, any novel payloads identified using our strategy will need to be evaluated for additional chemical features to ascertain their amenability for conjugation in ADC format.

It is crucial to note that in silico models may not encompass all biological intricacies. Thus, integrating these predictions with experimental validation is paramount. Validation of novel ADC targets and payloads typically includes cytotoxicity studies, binding affinity assays, and internalization assays, followed by animal models to assess tumor inhibition and safety profiles.

Our approach to identify the optimal target-indication-payload combination serves as a promising foundation for developing future insights, albeit requiring additional considerations related to the tumor microenvironment, tumor biology, linker and payload characteristics [79]. Building upon these insights and by leveraging additional data our future work will focus on identifying most effective combinations of target, linker and payload against a specific cancer type.

## Conclusions

We presented a list of clinically validated, as well as novel targets, for ADC development against a wide array of cancer indications. The findings underscore the significance of taking the mutational and genomic profile of target tumor type into consideration in order to provide precise and clinically effective targeting of ADCs. We extended our analysis to compile a list of potential payloads and initial exploration of target-indication-payload combination, which can provide guidance towards the development of ADC in a tumor targeted manner. The insights provided in our study can potentially improve the targeting of ADCs for specific patient populations and aid in guiding more effective clinical treatment responses.

## Materials and methods

In this section, the data acquisition and processing steps are described in detail.

### Identification of potential ADC target candidates

All protein coding genes (n = 20,090) were queried using the Human Protein Atlas (HPA) database version 22.0 with the goal to identify the membrane protein coding genes (n = 5543) as an initial filter (https://v22.proteinatlas.org/search/protein_class:Predicted+membrane+proteins). Subsequently, we utilized the HPA annotation to further narrow down the genes list. This led to exclusion of 668 genes with no evidence at protein level retaining 4875 genes exhibiting evidence at protein level. In the 3rd filter we retained genes (n = 1731) that did not show high expression in critical normal tissues (we considered a total of 13 tissues as critical normal tissue which is shown in Fig 2A) using the normal tissue data downloaded from the HPA download page (https://v22.proteinatlas.org/about/download). We calculated the percentage of samples with low, medium and high protein expression using the HPA IHC pathology dataset. And then as a proxy of protein expression levels, a quasi H-score (ranging between 0–300) was calculated using the following formula for remaining genes across 20 TCGA tumor types. Quasi H-score = (percentage of patients with low protein expression x 1) + (percentage of patients with medium protein expression x 2) (percentage of patients with high protein expression x 3). In order to keep the genes that show high expression in at-least 1 indication, we used 150 as a quasi H-score cutoff, which resulted in 763 genes. In the subsequent filtration stage, using the annotation provided by in silico human surfaceome [16] publicly available database (http://wlab.ethz.ch/surfaceome), only the 348 genes responsible for encoding the surface protein were considered for further analysis. We derived these initial steps as described by Razzaghdoust et al [14].

These 348 genes were further checked for consistency with other data types in the 6^th^ filtering step, which involves two sub-level filtering processes; 6a) Consistency between mRNA levels and IHC (Immunohistochemistry) data. TCGA Pan-Cancer (PANCAN) data from Xenabrowser [80] having FPKM mRNA expression levels across different TCGA cohorts, and RNA HPA as well as RNA GTEx tissue gene data from HPA (https://v22.proteinatlas.org/about/download) were used for this step. We verified consistency through two methods one using direct mRNA expression levels and another using description mentioned in the HPA database. In order to check the consistency using mRNA expression levels, we used quartiles to classify expression levels into four categories (not detected, low, medium and high) to match with IHC annotation. Expression levels of zero are categorized as not detected, expression levels between zero and first quartile are categorized as low, expression levels higher than first quartile but lower than third quartile as medium and expression higher than third quartile as high.The targets for which the expression levels are aligned in both datasets mRNA expression based calculated categories and IHC based expression levels from HPA database were considered consistent. 6b) Correlation of protein expression derived quasi H-score and TCGA mRNA expression derived quasi H-score. For this step, similar to quasi H-score calculation using protein expression data, we calculated quasi H-score using mRNA expression FPKM values. Samples with expression level less than first quartile were considered to be low expression, while samples with expression level higher than third quartile were considered to be high and samples with expression levels between first and third quartile were considered to be medium expression levels. Based on this quasi H-score was calculated using mRNA FPKM values. Genes scoring higher than 150 quasi H-score in both datasets (protein expression derived and mRNA expression derived) were chosen for further analysis.

Only 123 genes passed through this filtering process. In the subsequent step we used data from the GSE42519 study [15] in order to identify and remove the genes that are highly expressed in the HSCs and MPPs. The GSE42519 study covers microarray expression profiling data on normal cell landscape for the myeloid arm of the hematopoietic system. We used entire gene expression data to identify the first and third quartile in order to classify the samples expressing high, medium and low levels. In the last step, we annotated the genes using five criterias for evidence based filtering, 1) Literature: targets for which there is existing literature evidence elucidating their potential role in tumor biology. 2) Antibody: targets against which antibodies have been generated 3) Protein family targets, belong to a protein family where other proteins isoforms of which have been employed for the advancement of ADC in either clinical or preclinical setting 4) Preclinical: targets tested in preclinical setting 5) Clinical: targets tested in clinical setting. We filtered out genes without any annotations / evidence for any of the five criteria, resulting in 82 prioritized ADC targets. The overview of the entire approach is shown in Fig 1.

### Exploring the impact of mutated genes on the expression levels of prioritized ADC targets

We used the TCGA pan cancer mutation data downloaded from the Xenabrowser hub cohort named “TCGA Pan-Cancer (PANCAN)” [80]. The mutation data was generated under the MC3 project [81]. For the expression, TPM values were downloaded from the same Pan-Cancer cohort. We annotated the data using annotation files given in the above mentioned cohort from Xenabrowser. The names of the cancer types from the HPA data analysis were matched with the Pancan mutation data, considering 22 TCGA tumor subtypes. We used 416 mutated genes [8, 49] to query the expression level of 82 prioritized ADC targets identified using our screening method across 22 tumor subtypes. For the comparison of mutation vs wildtype group, we used wilcoxon test (non-parametric) and considered p value of 0.05 to find significant differences. In order to identify the strong association, we considered only the target with > = 1 log2 fold change and the cancer subtype having > = 5% population change by given mutation.

### Identification of potent tumor selective payload candidates

Developmental Therapeutics Program (DTP) from NCI60 has sensitivity data on more than 50K compounds. We downloaded the data from the NCI-DTP portal [82] covering 56,920 compounds with unique NSC ID numbers. There were many compounds having unique NSC ID, but mapping to the same compound name, therefore we removed the duplicate names and ended up with 47,310 total compounds. First we grouped these compounds into 2 categories, a) compounds having sensitivity in the picomolar (<1nM) range and b) compounds having sensitivity in the low nanomolar range (1nM - 10nM). Each category was passed through further filtering where we only retained the compounds having >50% response in at-least 1/9 indications in the NCI60 dataset. Subsequently the compounds tagged with failed NCI60 screening were eliminated resulting in 116 compounds in picomolar range and 797 compounds in low nanomolar range category. At this point, we established three distinct groups-1) compounds (n = 33) exhibiting sensitivity in only picomolar range b) compounds (n = 631) exhibiting sensitivity only low nanomolar range and c) compounds (n = 65) exhibiting overlapping sensitivity with both picomolar as well as low nanomolar range across 9 cancer indications covered by NCI60. Our analysis led to a total of 729 unique compounds. Additional annotations of these compounds were done for mechanism of action (MoA) and their clinical utilization as ADC payload. We further applied hierarchical clustering to identify similar or contrasting sensitivity patterns within these groups of compounds.

### Identification of target-indication-payload combination

In order to identify the prioritized suitable target-indication-payload combination, we first aligned prioritized target-indication data with payload-indication data derived from NCI60. In the next step, we mapped indications which exhibited 100% sensitivity against selected clinically tested ADC payloads (Dxd, exatecan mesylate, maytansine, monomethyl auristatin E, maytansine deriv, eribulin mesylate). We expanded the analysis by incorporating the impact of mutations on any of those resultant target antigens. The method outlined in the preceding section was used to find any significant (0.05 p value as cutoff) association with target antigen expression levels and gene mutations.

The entire analysis was done in IDE RStudio version 1.4.1106 using R version 4.1.0

## Supporting information

S1 Fig Heatmap of the compounds with low nanomolar range of sensitivity. Heatmap represents clustering of 631 compounds with sensitivity ranging between 1nM to 10nM across 9 indications from NCI60.
(TIFF)

S1 File Expression levels of 94 genes in hematopoietic stem cells (HSCs) and multipotent progenitor cells (MMPs). Expression levels of 94 genes in HSCs and MMPs.
(XLSX)

S2 File Expression levels of prioritized targets in normal tissues. Expression levels of 82 prioritized targets in 44 normal tissues. This data is used to generate the Fig 2A.
(XLSX)

S3 File Expression levels of prioritized targets in tumor tissues. Expression levels of 82 targets in 20 tumor tissues. This data is used to generate the Fig 2B.
(XLS)

S4 File TCGA tumor types abbreviations. TCGA tumor type abbreviations and their corresponding full names.
(XLSX)

S5 File Details of sensitivity and annotations for compounds within a low nanomolar range. Compounds with sensitivity ranging between 1nM to 10nM across 9 indications from NCI60 with their annotations. This data is used to generate figure provided in S1 Fig.
(XLS)

S6 File Heatmap of the compounds with sensitivity ranging between picomolar to ≦ 10nM. Compounds with sensitivity ranging between picomolar to ≦ 10nM (compounds exhibiting overlapping sensitivity with both picomolar as well as low nanomolar range across 9 NCI60 cancer indications). This data is used to generate Fig 9.
(XLS)

S7 File Target-indication-payload combination coupled with mutation association. Details regarding combination of potential target antigens, indications and clinically tested ADC payloads along with impact of gene mutation on expression level of target antigens.
(XLSX)

## Abbreviations

ADC: Antibody Drug Conjugate
NCI: National Cancer Institute
RADR^®^: Response Algorithm for Drug positioning and Rescue
DTP: Developmental Therapeutics Program
IHC: Immunohistochemistry
HSC: Hematopoietic Stem Cells
MPP: Multipotent Progenitor Cells
GEO: Gene Expression Omnibus
TCGA: The Cancer Genome Atlas
