# Supplementary figures and images for: Expanding the repertoire of Antibody Drug Conjugate (ADC) targets with improved tumor selectivity and range of potent payloads through in-silico analysis
Source: PLoS One. 2024 Aug 26;19(8):e0308604. doi: 10.1371/journal.pone.0308604 (PMC11346940; doi:10.1371/journal.pone.0308604)

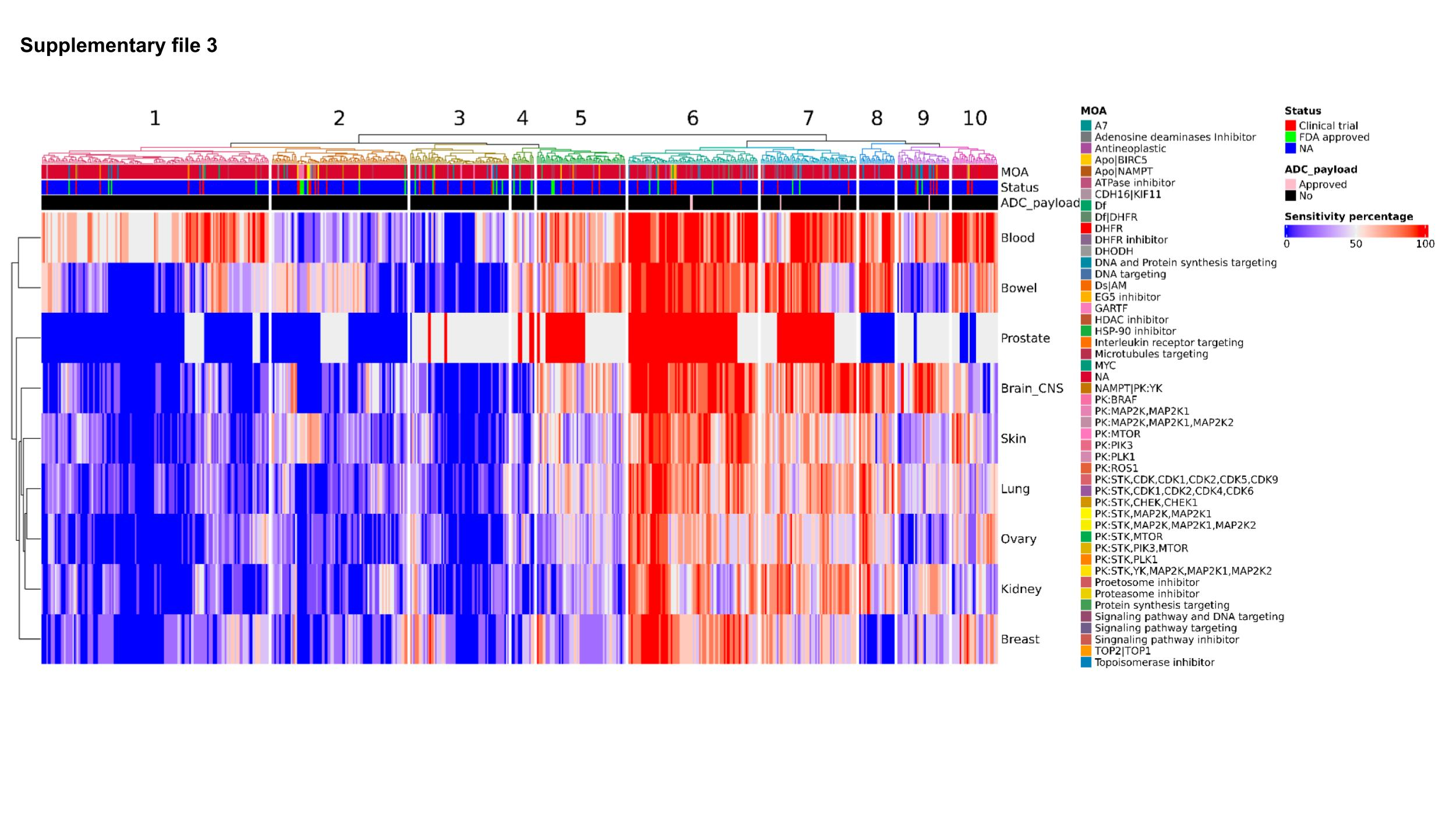

Supplement: S1 Fig — Heatmap represents clustering of 631 compounds with sensitivity ranging between 1nM to 10nM across 9 indications from NCI60. (TIFF) [file pone.0308604.s001.tiff]
